# A comparison of loop and split stoma for the management of various congenital and acquired gastrointestinal disorders requiring colostomy: An analysis of complications and outcomes

**DOI:** 10.12669/pjms.41.10.12083

**Published:** 2025-10

**Authors:** Erol Basuguy, Mehmet Hanifi Okur, Serkan Arslan, Hayrettin Ozturk, Bahattin Aydogdu, Mustafa Azizoglu

**Affiliations:** 1 Erol Basuguy, Department of Pediatric Surgery, Dicle University, Medical School, Diyarbakir/Turkiye; 2 Mehmet Hanifi Okur, Department of Pediatric Surgery, Dicle University, Medical School, Diyarbakir/Turkiye; 3 Serkan Arslan, Department of Pediatric Surgery, Dicle University, Medical School, Diyarbakir/Turkiye; 4 Hayrettin Ozturk, Department of Pediatric Surgery, Bezmialem University, Medical School, Istanbul/Turkiye; 5 Bahattin Aydogdu, Department of Pediatric Surgery, Dicle University, Medical School, Diyarbakir/Turkiye; 6 Mustafa Azizoglu, Department of Pediatric Surgery, Esenyurt Necmi Kadioglu State Hospital, Istanbul, Turkey

**Keywords:** Loop colostomy, Divided colostomy, Split colostomy, Colostomy complication, Outcomes

## Abstract

**Objective:**

The study aimed to compare the outcomes and complications of loop colostomy (LC) and split colostomy (SC) in pediatric patients requiring colostomy for various congenital and acquired gastrointestinal disorders. Additionally, we also wanted to evaluate the advantages and disadvantages of each technique and determine their role in contemporary clinical practice.

**Methodology:**

This study includes patients at the Department of Pediatric Surgery, Dicle University Faculty of Medicine, between January 2010 to January 2025. A total of 589 patients were analyzed, with data collected on demographics, stoma type (loop or split), associated complications and clinical outcomes. Patients were categorized into two groups: Group-I (loop stoma) and Group-II (split stoma) and the findings were compared statistically.

**Results:**

The most common indications for stoma creation were anorectal malformations (43.8%), Hirschsprung’s disease (24.3%), gastrointestinal perforation (13.8%) and necrotizing enterocolitis (6.3%). Of the 589 patients, 59.3% had loop stomas and 40.7% had split stomas. Common complications included excoriation (11.2%) and wound dehiscence (10.5%). Loop stomas had higher rates of prolapse (16%), evisceration (2%) and overall complications (38%), while split stomas showed higher rates of excoriation (14%) and wound dehiscence (14%). Stomas in the small intestine had higher rates of skin-related complications, while those in the sigmoid colon had a higher rate of stricture (3%).

**Conclusion:**

Both loop and split stomas are effective for managing pediatric gastrointestinal disorders, but each technique carries its own risk profile. Loop colostomies are more prone to prolapse and other internal complications, while split colostomies tend to have more skin-related issues. The choice of stoma type should be tailored to each patient’s specific clinical condition and anatomy.

## INTRODUCTION

If restoring intestinal continuity is not feasible, a colostomy-whether temporary or permanent-can be considered as an alternative and is widely used in pediatric surgical practice. In neonates and children, colostomy plays a vital role in managing both congenital and acquired gastrointestinal disorders.^1^,^2^

Although colostomies are regarded as protective and potentially life-saving, they come with significant risks, with morbidity and mortality rates varying depending on the surgical technique employed and the patient’s overall health.^3^ The creation of a stoma is associated with morbidity rates ranging from 21% to 60%, emphasizing the necessity for improved surgical approaches.^4^ In managing gastrointestinal conditions requiring colostomies, two primary techniques are commonly performed: loop colostomy (LC) and split colostomy (SC).^3^

Loop transverse colostomies, typically placed in the right hypochondrium, often result in a large stoma that is susceptible to complications such as prolapse, bleeding and difficulties in flushing the distal limb. These complications make post-operative management challenging and reduce patient tolerance.^5^,^6^ While loop ileostomies have become the preferred option for many surgeons, they still pose issues such as high liquid output, the need for intensive care and skin-related complications.^6^,^7^ A split colostomy is a procedure in which bowel continuity is completely disrupted, irrespective of the distance between the proximal and distal ends of the stoma.^8^ Furthermore, SC has been suggested as a safer and more effective alternative, with a lower incidence of stomal complications compared to traditional methods for diverting the distal colon.^4^

This study aimed to assess the advantages and complications of LCs, a commonly used stoma in various gastrointestinal pathologies requiring colostomy. By comparing LC with SC, we sought to determine their respective roles in contemporary clinical practice.

## METHODOLOGY

This study is a single-center retrospective analysis conducted at the Department of Pediatric Surgery, Dicle University Faculty of Medicine between January 2010 to January 2025. Patient records were reviewed retrospectively.Patients were divided into two groups: Group-I (loop colostomy) and Group-II (split colostomy). We evaluated patients based on age, gender, the location of the ostomy (e.g., ileum, colon, sigmoid colon), type of ostomy and associated complications.

### Ethical approval:

It was received from Dicle University Ethics Committee (Date: 2025/03/04. No: 86).

### Definitions:

- ***Loop Colostomy:*** A procedure in which a loop of the bowel is brought to the surface as a stoma without fully dividing the intestinal wall, resulting in a single stoma opening on the skin.
- ***Split Colostomy:*** A procedure where the bowel loop is fully divided, creating two separate stoma openings on the skin, each with its own incision.

### Inclusion Criteria:

- Patients aged 0-18 years, diagnosed and treated for stoma creation at the Pediatric Surgery Clinic of Dicle University Faculty of Medicine, were included in this study. Only patients who underwent stoma creation for any reason and whose records and data were complete and consistent in retrospective screening were included.

### Exclusion Criteria:

- Patients whose records and data were incomplete or inconsistent during the retrospective screening were excluded from the study.

### Statistical analysis:

Statistical analysis was performed using descriptive statistics, frequencies and other features for all categories. Continuous data are expressed as mean ± standard deviation. Shapiro-Wilk and Kolmogorov-Smirnov tests were used to assess the normality of continuous variables. For variables with a normal distribution, Student’s T-test was used for comparisons. Non-parametric tests were applied when the data were not normally distributed. Categorical variables were compared using the chi-square test. Statistical analysis was conducted using SPSS Statistics for Windows, Version 24.0 (IBM Corp, Armonk, NY, USA). A p-value of less than 0.05 was considered statistically significant.

## RESULTS

A total of 589 patients were included in this study, with 58.7% (n=346) being male. The median age was nine days (range: 2-180 days). The most common indications for stoma creation were anorectal malformation (43.8%, n=258), Hirschsprung’s disease (24.3%, n=143), gastrointestinal perforation (13.8%, n=81) and necrotizing enterocolitis (6.3%, n=37). Loop stomas were performed in 59.3% (n=349) of cases, while split stomas were performed in 40.7% (n=240) of cases. Of the stomas, 22.1% (n=130) were located in the small intestine and 77.9% (n=459) were in the colon. Among the colonic stomas, 54% (n=248) were situated in the sigmoid colon and 46% (n=211) in the transverse colon. The general characteristics of the study population are shown in Table-I.

**Table-I:** General characteristics.

Characteristics	N (%)
Age (day / min-max)	9 (2-180)
Gender (M/F)	346/243 (58.7% / 42.3%)
***Diseases***	
Anorectal malformation	258 (43.8%)
Hirschsprung disease	143 (24.3%)
GIS perforation	81 (13.8%)
Necrotizing enterocolitis	37 (6.3%)
Intestinal obstruction	27 (4.6%)
Intestinal atresia	18 (3.1%)
Perineal trauma	15 (2.5%)
Other traumas	7 (1.2%)
Volvulus	1 (0.2%)
Burn	1 (0.2%)
Gastrochisis	1 (0.2%)
***Stoma types***	
Loop stoma	349 (59.3%)
Split stoma	240 (40.7%)
***Stoma location***	
Small intestine	130 (22.1%)
Transvers colon	211 (35.8%)
Sigmoid colon	248 (42.1%)

The most common complications associated with stomas were excoriation (11.2%), followed by wound dehiscence (10.5%). A total of 33.3% (n=196) of patients experienced at least one complication. All complications are detailed in Table-II.

**Table-II:** Complications.

Complications	N (%)
Excoriation	66 (11.2%)
Wound dehiscence	62 (10.5%)
Prolapse	58 (9.8%)
Bridge ileus	23 (3.9%)
Perforation	13 (2.2%)
Bleeding	11 (1.9%)
Stricture	8 (1.6%)
Evisceration	7 (1.2%)
Torsion	3 (0.5%)
Overall complications	196 (33.3%)

When comparing stoma types, excoriation (14%) and wound dehiscence (14%) were more frequent in the split stoma Group-(*p*=0.049 and *p*=0.014, respectively). On the other hand, prolapse (16%), evisceration (2%) and overall complications (38%) were more common in the loop stoma Group (*p*=0.000, *p*=0.047 and *p*=0.001, respectively). No significant differences were observed in terms of torsion, bleeding, perforation, or stricture (Table-III).

**Table-III:** Comparison between stoma types.

Complications	Split stoma N (%)	Loop stoma N (%)	Total N (%)	p
Excoriation	33 (14%)	33 (9%)	66 (11%)	0.049*
Wound dehiscence	33 (14%)	29(8%)	62 (11%)	0.014*
Prolapse	0 (0%)	58 (16%)	58 (10%)	0.000*
Bridge ileus, Adhesion	8 (3.9%)	15 (3.9%)	23 (3.9%)	0.681
Perforation	3 (1%)	10 (3%)	13 (2%)	0.237
Bleeding	6 (3%)	5 (1%)	11 (2%)	0.353
Stricture	4 (2%)	4 (1%)	8 (1%)	0.717
Evisceration	0 (0%)	7 (2%)	7 (1%)	0.047*
Torsion	0 (0%)	3 (1%)	3 (0.5%)	0.286
Overall complications	58 (25%)	138 (38%)	196 (33%)	0.001*

* Less than 0.05 P value statistically significant.

Regarding stoma location, excoriation (22%), adhesion (9%) and wound dehiscence (22%) were more frequent in proximal stomas (small intestine) than in distal stomas (transverse, sigmoid colon) (*p*=0.000, *p*=0.002 and *p*=0.000, respectively). The rate of stricture (3%) was higher in stomas constructed at the sigmoid colon level (*p*=0.030). Prolapse (20%) was more common in stomas constructed at the transverse colon level (*p*=0.000). Overall complication rates were 42% for stomas in the small intestine, 42% for those in the transverse colon and 21% for those in the sigmoid colon (*p*=0.000) (Table-IV). The overall mortality rate was 4% (n=21).

**Table-IV:** Complication analysis in terms of stoma levels.

Complications	Small intestine (n=130) N (%)	Transvers colon (n=211) N (%)	Sigmoid colon (n=248) N (%)	Total N (%)	p
Excoriation	29 (22%)	25 (12%)	12 (5%)	66 (11%)	0.000*
Wound dehiscence	29 (22%)	23 (11%)	10 (4%)	62 (11%)	0.000*
Prolapse	6 (5%)	45 (20%)	9 (4%)	58 (10%)	0.000*
Bridge ileus	12 (9%)	4 (2%)	7 (3%)	23 (4%)	0.002*
Perforation	6 (1%)	2 (3%)	5 (3%)	13 (2%)	0.079
Bleeding	0 (0%)	4 (2%)	7 (3%)	11 (2%)	0.157
Stricture	0 (0%)	1 (1%)	7 (1%)	8 (1%)	0.030*
Evisceration	2 (2%)	3 (1%)	2 (1%)	7 (1%)	0.763
Torsion	1 (1%)	1 (0.5%)	1 (0.5%)	3 (0.5%)	0.890
Overall complications	55 (42%)	89 (42%)	52 (21%)	196(33%)	0.000*

* Less than 0.05 P value statistically significant.

## DISCUSSION

In our study, we observed a diverse patient population with various underlying conditions requiring stoma formation, including anorectal malformations, Hirschsprung’s disease, gastrointestinal perforations and necrotizing enterocolitis, among others. These findings align with existing literature, which emphasizes the broad spectrum of conditions for which stoma surgery is necessary in pediatric patients.^1^,^2^ In this study, we compared loop transverse colostomy (LTC) and split transverse colostomy (STC) in pediatric patients to evaluate their complication rates and clinical outcomes. Our findings revealed significant differences between these two techniques, reinforcing the need for careful consideration when selecting the most appropriate colostomy type for each patient.

Loop stomas, particularly those constructed in the transverse colon, are more prone to prolapse and evisceration. These findings in our study align with prior research, which has highlighted the increased risk of stoma prolapse in loop colostomies due to the incomplete division of the intestinal continuity.^3^ These complications arise due to the large size of the stoma, which may make it difficult to manage and lead to stoma dysfunction.^1^,^2^,^9^,^10^

Furthermore, loop stomas, being open at both ends, can lead to issues with distal limb flushing and increase the risk of dehydration and electrolyte imbalances due to high-output diarrhea.^2^,^7^,^11^ In contrast, split stomas, which have smaller openings, tend to be more prone to skin complications, such as excoriation and irritation, due to friction at the stoma site. These findings align with the increased rate of skin complications observed in our split stoma group. Additionally, split colostomies have been reported to have lower prolapse rates but an increased incidence of wound complications.^12^ A study by Oda et al.^8^ found that split colostomies had superior long-term outcomes regarding stoma function and complication rates compared to loop colostomies. However, they also highlighted the technical difficulties involved in the closure of split stomas, which require additional surgical expertise.^13^

In another study in the literature on loop ileostomies by Hallbook et al.^7^ it was emphasized that loop ostomies are easier to reverse but are more prone to complications such as dehydration and high fluid output. This supports our finding that LTC, despite being commonly performed, carries a significant burden of complications. Moreover, a recent meta-analysis by Gerçel et al.^3^ underscored the importance of patient selection, advocating for a tailored approach in choosing the most appropriate colostomy technique based on individual risk factors.

A particularly important aspect of our study was the examination of stoma location and its association with different complication rates. The overall complication rate observed in this study (33.3%) is consistent with prior literature, where complication rates have ranged from 21% to 60% depending on surgical technique and patient factors.^2^ We found that stomas located in the small intestine (proximal stomas) had higher rates of excoriation (22%), adhesion (9%) and wound dehiscence (22%) compared to those located in the distal colon (transverse and sigmoid colon). This finding is consistent with clinical studies indicating that small intestinal stomas tend to have higher rates of skin irritation and dehydration due to the higher fluid content of the small intestinal outlet^7^,^14^-^17^ Additionally, our study identified wound dehiscence as a common issue, particularly in STC, which may be attributed to the larger wound burden associated with the complete transection of the bowel.

In contrast, stomas constructed in the sigmoid colon were associated with a higher rate of stricture (3%), which aligns with studies suggesting that the sigmoid colon, with its narrower lumen, is more prone to stenosis and strictures after stoma formation.^16^ Additionally, stomas placed in the transverse colon have been found to be more prone to prolapse, with reported prolapse rates ranging from 2% to 27%, with an average of approximately 14%,^18^ reflected by the 20% prolapse rate observed in our study. We would also like to point out here that although transverse column stomas are easier to construct, they are more likely to cause complications such as prolapse due to their anatomical location and the tension applied to the stoma.

Our findings are consistent with many other studies in the field of pediatric stoma formation, which emphasize the need for a tailored approach depending on the patient’s underlying pathology and the stoma type. As in the study by Oda et al,^8^ emphasizing the balance between the technical ease of loop stomas and the lower complication rates of split stomas, it may be appropriate for surgeons to choose the most appropriate stoma type according to patient-specific factors rather than a single-type approach. Moreover, a systematic review by Gerçel et al.^3^ indicated the importance of prospective studies to further elucidate the long-term outcomes of different stoma techniques, as well as the impact of factors like stoma size, location and the underlying condition on postoperative recovery.

The mortality rate in our cohort was 4%, which, while relatively low, remains significant. Previous studies on pediatric colostomies have reported mortality rates ranging from 2% to 10%, influenced by factors such as the underlying condition and comorbidities. For example, a study by Chandramouli et al.^19^ found a mortality rate of 1.8% among 56 children who underwent colostomy closure. The mortality rate in our study was predominantly associated with the severity of the underlying disease, rather than the stoma procedure itself. Neonates with necrotizing enterocolitis and Hirschsprung’s disease are particularly vulnerable to systemic infections and organ failure, which can increase the risk of mortality despite the creation of a stoma.

Furthermore, long-term outcomes, including quality of life, stoma closure and the need for further surgical intervention, are critical considerations for pediatric patients with colostomies. Studies have shown that while many patients eventually undergo stoma closure, the long-term psychosocial and physical challenges associated with living with a stoma are considerable. This highlights the importance of comprehensive post-operative care and psychological support for both the patient and their family.^14^-^17^

### Limitations:

Among the limitations of this study, it is possible that there may be some missing elements in some data and long-term follow-up results due to being a retrospective study. In future studies, it is recommended to collect more comprehensive data using a prospective design and evaluate long-term results. However, despite these limitations, the large number of patients in this study provides useful information in the comparison and evaluation of loop and split stomas.

## CONCLUSION

Both loop and split stomas are effective surgical techniques for managing a range of pediatric gastrointestinal disorders. However, each has its distinct set of complications and the choice between the two should be made on a case-by-case basis, taking into account the specific pathology, anatomical considerations and potential for complications. While loop colostomy remains widely utilized, its higher prolapse and overall complication rates warrant careful patient selection. Conversely, split colostomy, though associated with increased wound-related complications, may offer a viable alternative with lower prolapse risk. The most important potential difference in the results presented above in this study, which also differs from similar studies in the literature, is the diversity in the patient groups. This adds new information and results to the literature, while also contributing to the strength of the study. The study has significant clinical potential, as it demonstrates different approaches to different diseases in children requiring ostomy. Despite all this, further prospective studies should focus on refining surgical techniques and postoperative care strategies to further improve patient outcomes in pediatric colostomy management.
